# 

**Published:** 1889-03-09

**Authors:** 


### CONTENTS.

**The Student's "Reckoning Day."** - 355

**Turning Over New Leaves.** "A Life Register"—"People We Meet" - 356

**Breakdown of the Hospital System.** By B. Burford Rawlings - 357

**A Colonial Congress** - 358

**The Doctor's Pulpit.**—Man and His Enemies - 359

**In a Coal Mine with Miners.** By a New Hand - 360

**A Child's Reproof** - 361

**Notes and News** - 362

**Everybody's Page** - 364

**Annotations.**—March Dust—The Horse and His Murderers—"Horseflesh" to the Fore - 365

**Within the Wards.**—Recent Advances in Wound Dressing - 366

**False Impressions.** By Caroline Fothergill, Author of "An Enthusiast," "A Voice in the Wilderness," etc. - 367

**Military Graveyards and Epitaphs in India**—II. By an Old Indian - 368

**Glances at the Insane in Other Lands**—VI—Victoria - 369

**Scraps and Gleanings. Amusements and Relaxation** 370

### EXTRA SUPPLEMENT—THE NURSING MIRROR.

**En Passant.**—Scarborough Sisters—Stamford Nursing Society—Rest and Relaxation—The Physical Strain of Nursing—"Work and Leisure"—Anxious Nurses—Heroism lxxxix
**Nurse Eyre's Mistake** xc
**Notes from Australia** xci
**Medical Missionaries** xci
**Everybody's Opinion.**—York Home for Nurses—Be Cautious—The Combination of Private Nurses xcii
**Appointments. Vacancies** xcii
**Notes and Queries** xcii

